# Open-Source Molecular Docking and AI-Augmented Structure-Based Drug Design: Current Workflows, Challenges, and Opportunities

**DOI:** 10.3390/ijms27073302

**Published:** 2026-04-05

**Authors:** Faizul Azam, Suliman A. Almahmoud

**Affiliations:** Department of Pharmaceutical Chemistry and Pharmacognosy, College of Pharmacy, Qassim University, Buraydah 51452, Saudi Arabia; s.almahmoud@qu.edu.sa

**Keywords:** molecular docking, structure-based drug design, open-source software, virtual screening, artificial intelligence, reproducible workflows

## Abstract

Molecular docking is a foundational technique in computational drug discovery, widely used to generate binding hypotheses, prioritize compounds, and support target-selectivity studies. The continued growth of open-source docking resources, together with improvements in scoring functions, sampling strategies, and hardware acceleration, has substantially lowered barriers to teaching, early-stage hit identification, and reproducible research. Beyond standalone docking engines, the open-source ecosystem now encompasses browser-accessible tools, preparation and analysis utilities, integrative modeling platforms, and AI-augmented methods for pose prediction, rescoring, and virtual screening. These developments have made docking workflows more accessible, customizable, and transparent across diverse research settings. This review examines open-source docking from a workflow-centered perspective, spanning study design, structural-data acquisition, binding-site definition, receptor and ligand preparation, docking execution, and post-docking validation. It further evaluates how open AI methods are being incorporated into these stages to expand structural coverage, improve screening efficiency, and support contemporary structure-based drug design. Collectively, this review outlines a practical and evidence-based framework for the effective use of open-source docking and virtual-screening pipelines in modern drug discovery.

## 1. Introduction

**Figure 1:**
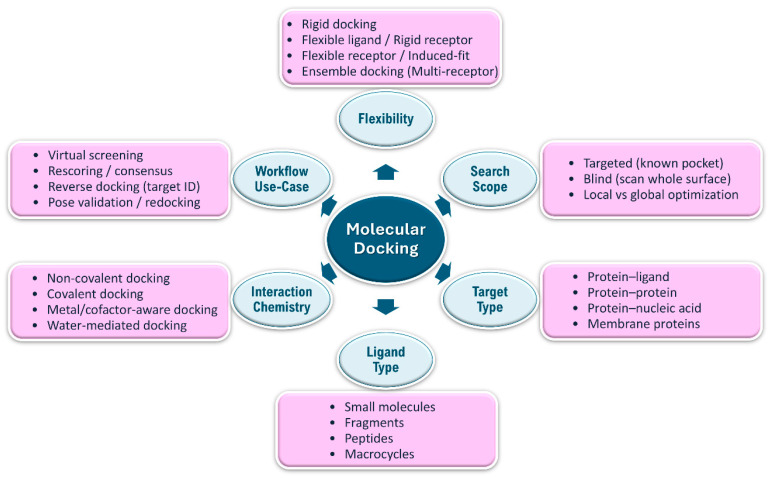
A conceptual map of molecular docking classification, encompassing flexibility, search scope, target and ligand types, interaction chemistry, and subsequent applications in structure-based workflows.

The field of Structure-Based Drug Design (SBDD) has made significant strides in the last twenty years, propelled by advancements in computational techniques and the swift growth of publicly available structural resources [1,2]. In particular, the Protein Data Bank (PDB) and the wider structural data ecosystem have facilitated access to experimentally determined macromolecular structures and numerous protein–ligand complexes, thereby aiding hypothesis-driven hit finding and lead optimization in academia and industry [3,4]. Simultaneously, the ability to simulate molecular interactions and predict binding affinities, once limited to pharmaceutical companies using costly proprietary software, has become accessible due to the rise in open-source and free academic tools, thereby reducing barriers to entry for structure-based workflows [5,6,7]. A key component of this evolution is molecular docking, a computational technique employed to predict the optimal orientation of one molecule relative to another when they interact to form a stable complex [8,9,10,11]. Figure 1 provides an overview of commonly used docking approaches and their applications. The main goal of molecular docking is to model the atomic interactions between a small molecule or ligand and a macromolecular target or receptor, predicting the binding conformation and the strength of the association in terms of affinity or score [12,13,14]. This process facilitates virtual screening (VS) of extensive chemical libraries, enabling researchers to prioritize a feasible number of compounds for experimental validation from a pool of millions or billions of candidates [15,16]. Traditional computational drug discovery has been influenced by the classical “lock-and-key” model, in which stochastic search algorithms and empirical scoring functions are used to estimate small-molecule binding [17].

The widespread use of docking in academic laboratories has been greatly facilitated by the availability of effective, open-source engines like AutoDock Vina, which offer improved throughput and usability compared to previous docking software generations [18,19]. Accessibility has been enhanced through web-based platforms that eliminate the need for installation and parameterization, offering standardized interfaces for docking jobs. Examples include SwissDock and blind-docking servers like CB-Dock2 [5,20,21]. The focus within the community has progressively transitioned from solely providing software access to prioritizing the quality of protocols, validation, and reproducible execution in diverse computing environments [22].

Recent advancements in AI technologies have begun to revolutionize traditional SBDD workflows at various stages, such as pose prediction, rescoring, and complex structure prediction [23]. AI co-folding and complex-prediction foundational models that deduce protein–ligand complex structures from sequence and ligand representations are of particular interest [24]. Notable examples are Uni-Mol (pose prediction and 3D molecular representation learning) [25], Umol (sequence-to-complex prediction) [26], Boltz-2 (complex prediction with affinity-oriented objectives) [27], and Chai-1 (multimodal biomolecular structure prediction) [28]. These approaches can produce high-quality initial geometries for subsequent modeling; however, current evidence indicates that they should be regarded as complementary to, rather than substitutes for, physics-based affinity estimation, especially when quantitative ranking is necessary [23,29]. Rigorous free-energy methods, such as alchemical approaches, can achieve high accuracy in certain contexts; however, they are relatively resource-intensive and consequently less prevalent in the initial screening phases [30,31,32,33].

The need for scalable and effective screening has intensified with the rapid growth of the make-on-demand chemical sector. Enamine REAL Space contains tens of billions of synthetically accessible compounds, highlighting how rapidly enumerated libraries can surpass the throughput of traditional single-workstation workflows [34,35]. As libraries expand to multi-billion scales, SBDD presents a significant data-engineering challenge alongside modeling challenges. This necessitates efficient storage formats and query tools, such as Parquet and DuckDB, to facilitate filtering, featurization, and orchestration in large-scale virtual screening pipelines [36]. These developments collectively offer a unified perspective on modern computer-assisted drug design and discovery that integrates open-source resources such as docking tools, accessible web services, specialized protocols, and emerging AI models into a coherent framework. Accordingly, this review aims to map the ecosystem that underpins these workflows.

## 2. Why Free Docking Tools and Resources Matter

**Figure 2:**
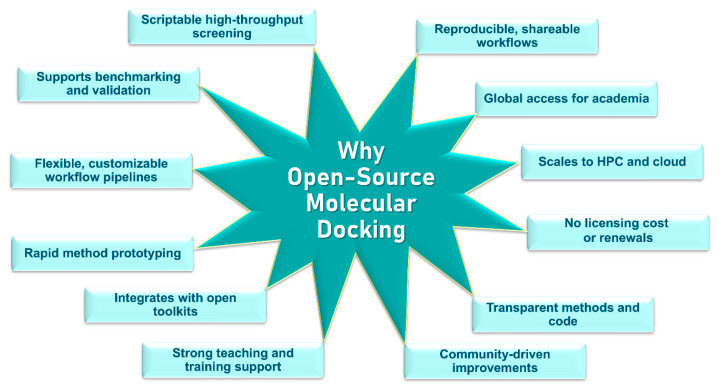
Rationale for open-source molecular docking tools.

The significance of free and open-source docking resources extends far beyond simple cost reduction; rather, it reflects a broader transition toward a scientific ecosystem that is more transparent, reproducible, and inclusive. Historically, computational chemistry and molecular modeling have been strongly influenced by proprietary software environments that require expensive licenses [37,38,39]. While such platforms have contributed significantly to the development of molecular modeling methodologies, their cost and restricted accessibility have often limited widespread academic adoption and hindered interoperability between research groups. In addition, the closed architecture of proprietary software can restrict researchers’ ability to inspect or modify the algorithms and parameterizations underlying computational predictions, thereby complicating transparent benchmarking and independent validation. The increasing availability of open docking frameworks and open data resources has therefore played an important role in expanding access to structure-based drug discovery and enabling collaborative methodological development across the global research community [22,40]. A flowchart summarizing the rationale for open-source molecular docking tools is shown in Figure 2.

A significant challenge in computational chemistry is the issue of reproducibility. Proprietary software typically operates as a black box, rendering the underlying algorithms and scoring functions unavailable for peer review. Open-source docking platforms address this limitation by offering transparent access to source code and methodological implementations, facilitating independent verification of computational workflows. This transparency enables researchers to assess algorithmic theories, replicate docking outcomes, and systematically compare various docking methodologies. This openness is recognized as crucial for ensuring methodological rigor and promoting the continuous enhancement of docking algorithms utilized in virtual screening and SBDD processes [22].

Moreover, financial accessibility represents a significant factor, highlighting the necessity of open computational tools. The licensing costs of commercial molecular modeling platforms can create significant financial obstacles for numerous academic laboratories, especially in developing regions or institutions with constrained research funding. Free docking software, open chemical databases, and publicly accessible web servers facilitate broader access to structure-based drug discovery technologies. Reducing financial barriers allows an increased number of researchers to engage in computational drug discovery and virtual screening initiatives [41]. This accessibility is especially significant for research focused on neglected or emerging diseases, where drug discovery efforts are frequently led by academic and public-sector laboratories instead of industry [42].

The value of free and openly accessible docking resources is also supported increasingly by prospective rather than purely retrospective evidence. In other words, these tools matter not only because they lower cost and improve transparency, but also because they have already contributed to experimentally validated hit discovery. For example, AlphaFold2-guided large-library docking with DOCK3.8 was used prospectively for ligand discovery against challenging membrane targets and showed that docking to predicted structures could recover hit rates and affinities comparable to those obtained with experimental receptor structures, with some identified ligands reaching low-nanomolar potency [43]. Likewise, the RosettaVS platform, while more accurately described as freely available for academic use rather than strictly open-source, identified prospective hits against KLHDC2 and NaV1.7 with reported hit rates of 14% and 44%, respectively, and the predicted binding mode for a KLHDC2 ligand was subsequently validated crystallographically [44]. Such studies are important because they demonstrate that open or freely accessible docking-centered workflows are no longer only methodological frameworks or teaching tools, but practical discovery engines capable of producing experimentally confirmed ligands when coupled to careful target preparation and downstream validation [43,44,45,46,47,48,49,50,51,52,53]. Few selected prospective success stories from free or academically accessible docking/SBDD workflows are summarized in Table 1.

Open computational infrastructures also play a crucial role in education and training within modern medicinal chemistry and cheminformatics. One of the challenges associated with advanced computer-aided drug design workflows is the steep learning curve required to master specialized computational tools and scripting environments. Educational initiatives based on open-source resources have addressed this limitation by providing accessible training frameworks that integrate theoretical concepts with practical computational exercises. Platforms such as TeachOpenCADD offer modular tutorials and reproducible workflows implemented in Python and interactive notebook environments, enabling students and early-career researchers to execute and modify realistic docking and virtual screening pipelines [54]. These open educational approaches have proven valuable for expanding computational training in medicinal chemistry curricula and for supporting remote or self-directed learning environments [55,56].

Finally, open-source docking tools offer flexibility and adaptability that are difficult to attain in static proprietary environments. The public availability of their source code allows researchers to modify and extend these tools to tackle emerging methodological challenges. Modern open frameworks make it easier to combine docking algorithms with machine-learning scoring functions and automated high-throughput screening workflows. Recent developments, such as modular docking frameworks in contemporary programming environments, demonstrate how open infrastructures facilitate rapid algorithmic innovation and integration with artificial intelligence methodologies [57,58]. Community-driven ecosystems are progressively facilitating large-scale virtual screening, advanced docking methodologies, and collaborative initiatives in the SBDD field [59,60,61].

## 3. Bibliometric Overview of Open and Commercial Docking Platforms

**Figure 3:**
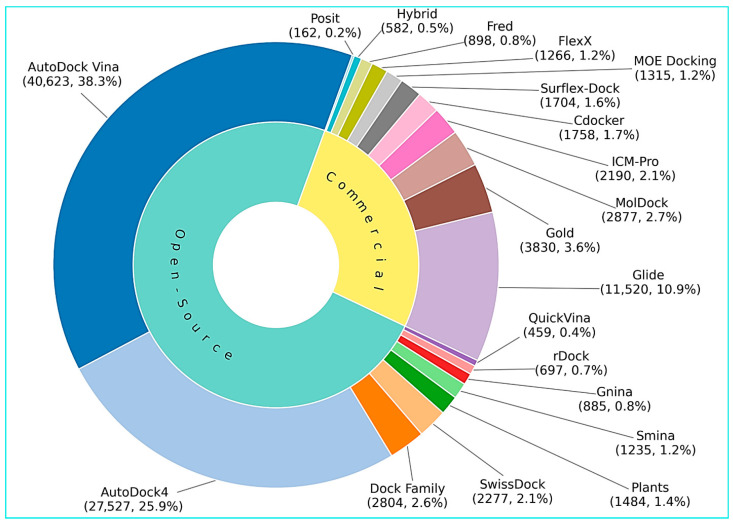
Sunburst chart summarizing representative open-source and commercial tools, with segment sizes proportional to Google Scholar citation counts as of 8 March 2026. Citation counts and their corresponding percentages are reported in parentheses. These counts are intended only as a rough proxy for historical visibility and community adoption and should not be interpreted as a direct measure of methodological superiority, predictive accuracy, or current practical performance.

Citation counts are an imperfect but still informative indicator of the visibility, dissemination, and historical uptake of computational tools within the scientific literature. In the docking and broader SBDD landscape, the present bibliometric snapshot suggests that open and freely accessible platforms have achieved wider representation in academic publishing than commercial alternatives. This pattern is driven most clearly by the exceptional prominence of the AutoDock family, especially AutoDock4 and AutoDock Vina, and is further reinforced by the continued citation visibility of other open tools such as the DOCK family, smina, GNINA, rDock, SwissDock, and related academic platforms. By contrast, commercial programs such as Glide, GOLD, MOE Docking, Discovery Studio/CDOCKER, Surflex-Dock, FlexX, and other proprietary suites remain highly influential and widely used, but their representation in the published literature appears, on the whole, to be less dominant than that of the most established open platforms. At the same time, these citation patterns should not be overinterpreted as evidence of methodological superiority. Citation frequency is shaped by multiple confounding factors, including software age, the prominence and accessibility of the original reference paper, differences in citation habits across disciplines, and the fact that commercial tools may be heavily used in industrial pipelines without generating proportionate citation records in the public literature. Moreover, citation counts do not directly capture docking accuracy, scoring reliability, maintenance activity, workflow integration, user support, or suitability for specific target classes and screening scenarios. Accordingly, Figure 3 should be interpreted primarily as a bibliometric overview of historical visibility and community adoption, not as a ranking of technical performance. Even so, the overall trend remains clear: the academic literature appears to give broader and more sustained visibility to open docking platforms, whereas commercial tools retain strong but comparatively more selective prominence, especially in well-funded academic and industrial settings.

## 4. Docking Workflow

### 4.1. Designing the Docking Study

**Figure 4:**
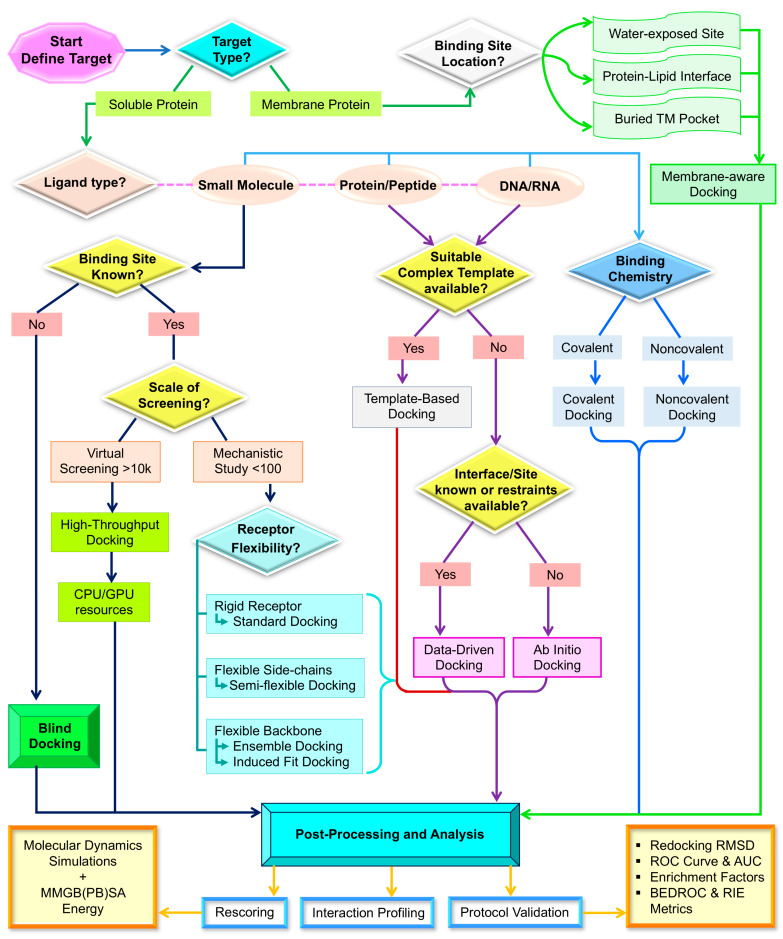
Decision workflow for selecting an appropriate docking strategy based on target class, membrane versus soluble protein, binding-site location ligand class, sampling requirements, receptor flexibility, and post-docking validation.

A docking study is most effective when it is designed as a structured decision-making framework that systematically aligns the research objective with the appropriate levels of conformational sampling, scoring, and validation, as outlined in Figure 4. Accordingly, the criteria for success should be defined in relation to the intended application, whether the goal is accurate pose prediction for mechanistic interpretation, early enrichment in virtual screening, or the assessment of selectivity trends across closely related targets. From this perspective, docking performance should not be regarded as an inherent attribute of a particular software platform. Instead, it reflects the combined influence of the search algorithm, scoring function, available experimental knowledge, and the extent to which the protocol has been validated using appropriate positive and negative controls [62,63,64].

In virtual screening, performance assessment should emphasize early recognition rather than global rank correlation because the practical value of a screening campaign lies in placing active compounds near the top of the ranked list. Metrics such as enrichment factor, BEDROC, and related early-recognition measures are therefore often more informative than global ROC-based summaries alone, although each metric has recognized limitations and should be interpreted in the context of dataset composition and decoy design. Benchmark construction is likewise not a minor reporting detail, as unrealistic active/decoy sets and chemically biased benchmarks can substantially overestimate apparent performance. For this reason, the selected metric, dataset design, and validation objective should be reported explicitly as part of methodological rigor [65,66].

### 4.2. Acquisition of Structural Data and Selection of Biological Assemblies

Whenever possible, structural coordinates should be derived from experimentally determined models deposited in the Protein Data Bank. In the absence of a suitable experimental structure, high-quality predictive models, including those generated by AlphaFold-class methods, may serve as useful alternatives, provided that their limitations are acknowledged. The selected structure should correspond to the biologically relevant assembly rather than merely the asymmetric unit, and it should retain any cofactors, catalytic ions, prosthetic groups, or tightly bound ligands that define the physicochemical environment of the binding site [3,67].

This choice is especially important because the docking target is not simply a protein scaffold, but a chemically specific recognition environment. Binding-site geometry, electrostatics, and accessibility may all change if an incorrect oligomeric state is chosen or if essential metals, cofactors, or bridging water molecules are omitted. These considerations are particularly critical for metalloenzymes and related systems in which coordination geometry contributes directly to molecular recognition and catalysis [68,69]. Key preparation and post-docking analysis tools that support reproducible workflows are summarized in Table 2.

### 4.3. Binding-Site Definition and Search-Space Setup

The most important practical aspect for small molecules is the knowledge of the binding site. When a co-crystallized ligand, catalytic residues, mutagenesis data, or biochemical restraints are available, they should guide the placement of the box center and the dimensions of the search space. Reporting the coordinates and size of the search region is essential for reproducibility, particularly in comparative studies, where differences in docking outcomes may otherwise reflect inconsistent box definitions rather than meaningful differences between docking engines [70].

This step warrants particular emphasis because the search space directly influences both pose prediction and screening performance. Overly narrow boxes can artificially improve apparent pose recovery by constraining sampling around the expected solution, whereas excessively large boxes reduce efficiency and increase the likelihood of irrelevant placements. Systematic studies using Vina have shown that search-space selection measurably affects both docking accuracy and enrichment [18]. In cases where the pocket is unidentified, employing a global strategy, such as blind docking or pocket discovery followed by focused docking, is suitable for generating hypotheses [71,72,73,74]. However, this approach should be regarded as exploratory and necessitates subsequent robust validation.

For membrane proteins, however, binding-site definition requires an additional level of classification based on the spatial relationship of the pocket to the lipid bilayer. A water-exposed site remains accessible from the extracellular or intracellular aqueous phase and therefore resembles a conventional soluble-protein pocket more closely, although its surrounding topology is still constrained by the transmembrane architecture. By contrast, a protein–lipid interface site is positioned on the membrane-facing surface of the receptor, often in an extrahelical groove or allosteric crevice, where ligand recognition is shaped not only by the protein surface but also by bilayer depth, local polarity, and lipid occupancy [75,76,77]. A buried transmembrane (TM) pocket lies within the helical bundle itself and includes many canonical orthosteric cavities in membrane receptors; in such cases, ligand binding may involve passage through vestibular or otherwise restricted access routes before the final bound conformation is achieved. This classification is not merely descriptive, because solvent exposure, residue composition, desolvation penalties, and ligand-access pathways differ substantially across these site types and can directly influence search-space definition, receptor preparation, and pose interpretation [78,79,80].

### 4.4. Introducing Receptor Flexibility and Chemistry-Gated Pathways

Once the binding site and, where relevant, its membrane context have been defined, receptor flexibility should be incorporated only to the extent supported by structural or biochemical evidence. A rigid receptor is often sufficient for large-scale screening and may even be preferable when the available structure already represents a suitable holo-like conformation. Limited side-chain flexibility may be appropriate when key residues are known to undergo local adjustments, whereas larger conformational changes may necessitate ensemble docking or induced-fit-like strategies [81,82]. Because these approaches increase computational cost and introduce a greater risk of overfitting, the meaning of receptor flexibility in a given protocol should be defined explicitly [83,84,85].

Interaction chemistry should likewise be treated as an early methodological decision rather than a late-stage refinement. Standard non-covalent docking assumptions are not appropriate for all ligand classes. Covalent ligands require methods that explicitly account for reactive warheads, residue compatibility, and reaction geometry. Reviews of covalent docking have emphasized that it should be considered a distinct methodological branch, and reactive docking approaches such as WIDOCK [86] illustrate how explicit treatment of warhead reactivity can improve both retrospective and prospective covalent screening [87,88,89].

### 4.5. Structural Quality Assessment Before Docking

Prior to initiating protein preparation, the selected structure should be evaluated for its suitability as a docking target. Relevant considerations include experimental resolution, missing loops, absent side chains, alternate conformations, engineered mutations, crystal-packing artifacts, and local disorder within the binding region. In docking applications, such features are particularly consequential because inaccuracies in the pocket region are often more detrimental than imperfections elsewhere in the structure. Structural quality assessment should therefore be treated as an explicit component of protocol design rather than as an informal preliminary check [90].

For predicted structures, local realism within the binding site is generally more important than global fold accuracy. Recent studies assessing AlphaFold-derived models as docking targets have shown that naive use of predicted structures can reduce docking and virtual-screening performance relative to experimentally determined structures, even when the overall backbone is highly accurate. These studies further suggest that post-processing, removal of low-confidence segments, or flexible treatment of local regions may improve performance, but do not eliminate the need for target-specific scrutiny. Accordingly, confidence assessment should focus primarily on the binding pocket rather than on whole-protein quality scores alone [91,92,93].

### 4.6. Ligand Preparation

The preparation of ligands determines the specific chemical forms of compounds evaluated in a screening workflow. It is advisable to keep the canonical chemical representations of compounds, like SMILES or SDF, distinct from docking-specific formats such as PDBQT. This separation facilitates the preservation of critical information, including stereochemistry, protonation history, and atom-typing assignments [90,94]. 

Open Babel remains a fundamental open-source toolkit for file conversion and routine cheminformatics tasks, frequently representing the most efficient choice for compound standardization in screening pipelines [95]. Gypsum-DL is a freely available tool designed for structure-based virtual screening, enabling the generation of screening-ready three-dimensional ligand libraries with configurable enumeration options [96].

Docking workflows utilizing the Vina family typically require the preparation of both ligand and receptor files in PDBQT format. Meeko provides a continuously updated open-source interface for the preparation of AutoDock-compatible inputs and the export of outputs in formats conducive to analysis, such as SDF, thereby minimizing file-format obstacles between docking and subsequent quality control or analysis [97]. The manuscript should present ligand preparation as a clear methodological policy decision. This includes detailing whether protomers, tautomers, and stereoisomers were enumerated, the number of conformers generated, and any filtering criteria applied before docking. The preparation choices for cross-study comparability are often more vital than the selection of closely related docking engines.

**Table 2:** Preparation and analysis tools for docking and structure-based drug design (SBDD).

SN	Tools	Stage	Primary Function	Typical Application	Reference/Official Link
1	AutoDockTools/MGLTools	Preparation + visualization	PDBQT preparation scripts, charge/atom typing, box setup, result viewing	AutoDock/Vina input preparation	[85] https://ccsb.scripps.edu/mgltools/
2	AutoSite	Binding-site prediction	Clusters high-affinity points to define pockets and pseudo-ligands	Pocket identification/box definition	[98] https://ccsb.scripps.edu/autosite/
3	BINANA	Interaction analysis	Geometry-based receptor–ligand interaction characterization	Post-docking contact classification	[99] https://github.com/durrantlab/binana
4	DeepPocket	Deep-learning pocket detection	3D CNN-based site detection and segmentation	Pocket prediction before blind/local docking	[100] https://github.com/devalab/DeepPocket
5	Dimorphite-DL	Protonation-state enumeration	Small-molecule ionization-state prediction	pH-aware ligand preparation	[101] https://github.com/durrantlab/dimorphite_dl
6	DockRMSD	Pose comparison/atom mapping	Graph-isomorphism-based symmetry-corrected RMSD	Benchmarking and pose-evaluation workflows	[102] https://aideepmed.com/DockRMSD/
7	Fpocket	Pocket detection	Voronoi tessellation/alpha-sphere cavity detection	Pocket finding and descriptor extraction	[103] https://github.com/Discngine/fpocket
8	Gypsum-DL	Ligand preparation	Enumerates ionization/tautomer/chirality/ring forms and builds 3D structures	Preparing docking-ready ligand libraries	[96] https://github.com/durrantlab/gypsum_dl
9	Meeko	Ligand/receptor preparation for AutoDock	Parameterization and PDBQT generation	Ligands, receptors, flexible side chains, nucleic acids	[97] https://meeko.readthedocs.io/
10	MolProbity/Reduce	Structure validation and hydrogen optimization	All-atom contact analysis and H placement	Protein/nucleic-acid validation before docking	[104] https://github.com/rlabduke/MolProbity
11	Molscrub	Ligand state enumeration	3D conformer generation, tautomer/protomer enumeration, pH correction	Preparing realistic ligand inputs for docking	https://github.com/forlilab/molscrub
12	ODDT	Cheminformatics/docking analysis toolkit	Unified Python toolkit for modeling, descriptors, interaction fingerprints, scoring	Post-processing, ML descriptors, docking analytics	[105] https://github.com/oddt/oddt
13	Open Babel	Ligand/receptor conversion and cleanup	Format conversion, protonation, 3D generation, atom typing	Interconversion across docking file formats	[95] https://openbabel.github.io/
14	Open-Source PyMOL	Visualization/analysis	Molecular visualization	Structure inspection, binding-mode analysis, figure preparation	https://github.com/schrodinger/pymol-open-source
15	P2Rank	Binding-site prediction	Machine-learning-based protein–ligand binding site prediction	Pocket prediction before docking	[106,107] https://github.com/rdk/p2rank
16	PacDOCK	Workflow/post-docking analysis	Conformation comparison, visualization, and clustering of docking results	Post-docking analysis and clustering	[108] https://pegasus.lbic.unibo.it/pacdock/
17	PDB2PQR	Receptor electrostatics preparation	Assigns charges/radii and creates PQR files	Protonation/electrostatics-aware receptor prep	[109] https://pdb2pqr.readthedocs.io/
18	PDBFixer	Receptor cleanup	Fixes missing atoms/residues, adds hydrogens/solvent-related corrections	Preparing imperfect PDB structures	[110] https://github.com/openmm/pdbfixer
19	pdb-tools	PDB file manipulation	Lightweight CLI editing of PDB structures	Chain selection, cleanup, renumbering, extraction	[111] https://github.com/haddocking/pdb-tools
20	PLIP	Interaction analysis	Rule-based detection and visualization of noncovalent protein–ligand contacts	Post-docking interaction profiling	[112] https://github.com/pharmai/plip
21	PoseBusters	Pose plausibility checks	Rule-based quality checks for generated/docked poses	Post-generation/post-docking QC	[113] https://github.com/maabuu/posebusters
22	PoseCheck	Complex quality analysis	Quality checks for generated protein–ligand complexes	Post-prediction QC and comparison	[114] https://github.com/cch1999/posecheck
23	PPM server (OPM)	Structure preparation	Membrane positioning/orientation	Preparing membrane protein targets before docking or other structure-based studies	[115] https://opm.phar.umich.edu/ppm_server
24	ProLIF	Interaction fingerprints	Protein–ligand interaction fingerprints from docking/MD/structures	Pose comparison and interaction-frequency analysis	[116] https://github.com/chemosim-lab/ProLIF
25	PyViewDock	Visualization	PyMOL docking-viewer plugin	Inspecting and browsing docking poses	https://github.com/unizar-flav/PyViewDock
26	RDKit	Ligand preparation/cheminformatics	Molecule standardization, descriptors, conformers, substructure logic	SMILES/SDF cleanup, enumeration, fingerprints, 3D conformers	https://www.rdkit.org/
27	Ringtail	Virtual-screening result management	SQLite-based storage, filtering, visualization for AutoDock/Vina outputs	Managing large docking campaigns	[117] https://github.com/forlilab/Ringtail
28	sPyRMSD	Pose comparison/RMSD	Symmetry-corrected RMSD in Python	Redocking evaluation and pose clustering	[118] https://github.com/RMeli/spyrmsd

### 4.7. Docking Simulations

Docking is most commonly introduced in the context of small-molecule binding to a defined receptor pocket, where the principal objective is to predict plausible binding poses and rank protein–ligand interactions within a relatively localized search space. In this conventional setting, docking performance depends strongly on binding-site definition, receptor and ligand preparation, sampling strategy, and scoring-function behavior [8,23]. As the size, flexibility, and physicochemical complexity of the interacting partners increase, docking problems become progressively less similar to the classical small-molecule case. For protein–protein, protein–peptide, and protein–nucleic acid docking, the central methodological question is therefore whether a suitable template or experimentally supported interface information is available. When restraints, templates, or biochemical priors exist, data-driven docking can substantially reduce the search space and improve interpretability. HADDOCK is a representative integrative framework in this context, as it explicitly incorporates experimental or predicted interaction information into model generation and refinement [119,120].

In the absence of such prior information, ab initio strategies become necessary, and clustering assumes particular importance because scoring functions alone are often insufficient to identify native-like models within a very large conformational landscape [119,120]. ClusPro exemplifies this principle in protein–protein docking, where cluster populations are central to model selection [121]. For peptide docking, Rosetta FlexPepDock [122] remains an important refinement framework because peptide flexibility and folding upon binding are often the dominant sources of complexity. For protein–DNA and protein–RNA systems, specialized tools such as NPDock [123] reflect the distinct electrostatic and shape-complementarity features of nucleic-acid interfaces and provide integrated workflows for docking, scoring, clustering, and refinement [124,125].

A related but distinct challenge arises when docking problems involve membrane-embedded targets, because in such systems, the bilayer is not merely a structural background but part of the recognition environment itself. When the relevant binding region is located at the protein–lipid interface or within a buried transmembrane environment, the membrane directly influences ligand orientation, access pathways, and local polarity. This point is methodologically important because many conventional docking programs were developed primarily for water-exposed cavities and may not adequately capture membrane depth preferences, lipid-facing polarity, or the bilayer-coupled orientation of lipophilic ligands. For membrane-associated chemotypes, extrahelical allosteric modulators, sterol-like ligands, or other bilayer-partitioning molecules, membrane-aware docking, or at minimum, membrane-informed receptor preparation and post-docking filtering, is therefore preferable to a purely aqueous interpretation of the binding site [77,78,79,80,126,127]. The currently available open-source/free for academic use software ecosystem is summarized in Table 3, and major free and web-accessible docking resources are listed in Table 4.

### 4.8. Docking Parameters, Sampling Settings, and Reproducibility

A reproducible docking study should report more than the name of the docking engine. At a minimum, the workflow should specify the software version, search exhaustiveness or equivalent sampling depth, number of poses retained, random-seed policy, treatment of receptor flexibility, search-space definition, scoring mode, and any non-default settings. These parameters are not incidental, as they determine the extent of conformational sampling and can materially affect both pose prediction and screening enrichment [18,195].

This level of reporting has become particularly important because many published docking studies remain difficult to reproduce or compare rigorously. Critical analyses of docking-based virtual screening have shown that apparent agreement across studies may conceal major differences in protocol design, validation practice, and data curation. Consequently, parameter reporting, preservation of input files, and transparent description of filtering and post-processing steps should be regarded as integral components of the core methodology rather than as supplementary details [196,197].

### 4.9. Post-Docking Validation and Potential Refinement

Post-docking analysis should combine at least three elements: assessment of pose plausibility, comparison among alternative poses or clusters, and validation against appropriate controls. In pose-prediction settings, redocking and cross-docking remain informative controls; in screening contexts, discrimination between active and inactive compounds, or between active compounds and decoys, is more relevant. Equally importantly, physically implausible solutions should be filtered even when they receive favorable docking scores. PoseBusters provides a practical example of this principle by evaluating stereochemical validity, steric clashes, bond geometry, ring planarity, and related criteria that conventional RMSD-based assessments may overlook [113].

When rescoring or refinement is applied, it should be presented as support for hypothesis refinement rather than as definitive affinity prediction. Machine-learning-assisted rescoring tools such as GNINA can improve ranking relative to purely empirical scoring in some settings [198], whereas molecular dynamics-based endpoint methods such as MM/PBSA and MM/GBSA may provide additional energetic interpretation after docking [199,200,201,202,203,204]. However, both classes of methods require calibration and careful interpretation. More recent benchmarking initiatives such as PoseBench further highlight that realistic tasks, including apo-to-holo docking and multi-ligand prediction, can expose limitations that are not evident in simplified redocking benchmarks [205].

### 4.10. Common Failure Modes and Limits of Interpretation

Docking results should ultimately be interpreted within the known limits of the method. High docking scores do not necessarily correspond to accurate binding affinities, and no single docking program performs best across all targets. Benchmarking studies have repeatedly shown that docking and screening power are generally more reliable than affinity ranking power, which remains a major weakness of most scoring functions. This means that docking is usually better suited for pose generation, prioritization, and hypothesis formation than for quantitative prediction of binding free energy [206,207,208,209]. Several recurring sources of overinterpretation should therefore be avoided. Small score differences between closely related ligands are often not meaningful; a visually attractive pose may still be chemically implausible if protonation, tautomerism, water mediation, or receptor conformation are misassigned; and a protocol validated only by redocking may fail in cross-docking, blind docking, or prospective screening. Docking should therefore be presented as one component of an evidence chain that is strengthened by structural data, mutagenesis, biochemical assays, orthogonal screening metrics, or more detailed simulation rather than as a stand-alone proof of mechanism [94,113,210,211,212,213].

## 5. AI-Augmented Structure-Based Drug Design

### 5.1. Conceptual Role of AI in SBDD

Artificial intelligence (AI) is continuously incorporated into SBDD as an augmentation rather than a complete substitute for physics-based modeling. In practical SBDD workflows, AI plays a significant role at three levels: the generation of target or complex structures when experimental models are incomplete or unavailable, the navigation of extensive chemical libraries prior to exhaustive docking, and the post-docking rescoring or affinity estimation. The methodological importance of AI is primarily in reorganizing the screening pipeline into a hierarchical process, rather than merely displacing docking. This involves integrating rapid, learned models with physics-based filtering and chemically informed validation. Recent perspectives on modern SBDD emphasize that the most effective workflows increasingly integrate data-driven and physics-based components, rather than viewing them as competing paradigms [1,32]. Representative AI-augmented open-source SBDD tools are outlined in Table 5. Recent comparative benchmarks now make clear that AI methods do not uniformly surpass classical docking across all settings. PoseBench showed that deep-learning co-folding methods often outperform conventional and deep-learning docking baselines in broadly applicable apo-to-holo prediction settings, yet remain challenged by new binding poses and by balancing structural accuracy with chemically faithful protein–ligand interaction patterns [206]. PoseX extended this picture to self-docking and cross-docking and reported that AI-based methods outperformed physics-based methods in overall docking success rate, while also showing that relaxation remains important for improving structural plausibility and that several co-folding models still suffer from ligand chirality errors [214]. By contrast, the recent Bento benchmark provides a more conditional view in pocket-aware, drug-design-relevant settings: classical and deep-learning docking methods were often comparable on regular drug-like ligands, co-folding methods were more clearly advantageous for structurally complex ligands, physics-based methods retained practical speed advantages, and all tested approaches generalized poorly to unseen pockets [215]. Accordingly, the most important methodological question is not whether AI is globally superior to classical docking, but which model class is most appropriate for a specific structural regime and decision point within the SBDD workflow.

### 5.2. Structural Availability and AI-Assisted Model Generation

**Figure 5:**
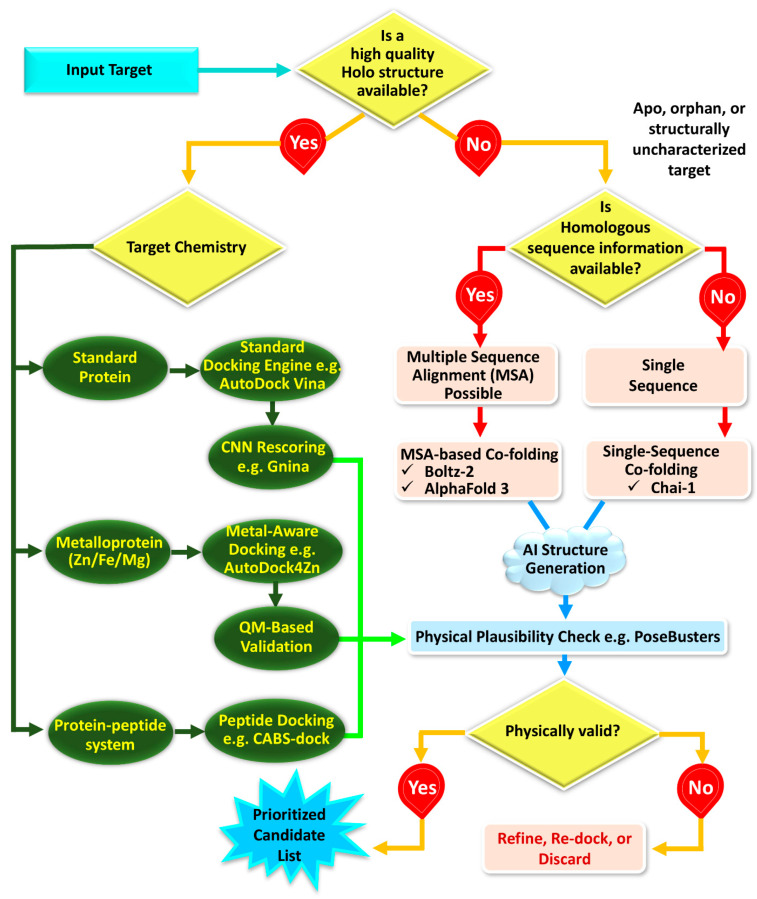
Decision workflow for AI-augmented structure-based drug design, beginning with structural availability, branching through AI-based structure generation when holo complexes are unavailable, and ending with target-class-specific docking and physical validity assessment.

The initial decision point in an AI-augmented SBDD workflow involves determining the availability of a high-quality holo structure (Figure 5). In the presence of such a structure, conventional docking serves as the methodological baseline, with AI being most effectively integrated downstream via rescoring, rank fusion, or prioritization. In the absence of suitable experimental complexes, recent co-folding and all-atom structure-prediction models offer a viable alternative for constructing initial receptor–ligand hypotheses. AlphaFold3 has shown that it is possible to jointly predict complexes that include proteins, nucleic acids, small molecules, ions, and modified residues within a single model framework [216]. Meanwhile, RoseTTAFold all-atom has expanded these concepts to encompass generalized biomolecular modeling and design [217]. Open tools such as Boltz-1 [218] and Chai-1 [28] expand access to co-folding-style workflows. The preprint detailing Boltz-2 indicates that foundation models may increasingly integrate structure prediction with affinity estimation [27]. Because these methods are supported wholly or partly by preprint-stage literature, their reported performance should be interpreted cautiously and regarded as provisional pending broader independent validation and peer-reviewed assessment. Accordingly, they are best viewed here as promising emerging developments rather than fully established standards in AI-augmented SBDD.

These advancements are particularly significant for apo targets, orphan targets, and novel proteins lacking ligand-bound structures. At the same time, AI-generated complexes should still be treated as candidate hypotheses rather than validated binding modes. Recent PoseBench, PoseX and Bento benchmarks support the use of AI structure generation primarily as a hypothesis-enabling layer for difficult apo, cross-docking, or high-flexibility scenarios, not as a universal replacement for experimentally anchored docking protocols [205,214,215].

### 5.3. AI-Enabled Navigation of Ultra-Large Chemical Space

AI significantly contributes to modern SBDD by facilitating an examination of chemical space before the docking process. The expansion of make-on-demand libraries from millions to billions of compounds has rendered naïve exhaustive docking increasingly challenging to implement in standard academic workflows. Extensive prospective studies indicate that increasing library size can uncover novel chemotypes and enhance hit discovery; however, this necessitates efficient library triage as an operational requirement. Vector-based molecular representations and approximate nearest-neighbor indexing provide an effective means to minimize the search space prior to the implementation of more resource-intensive physics-based methods. Embeddings produced by graph neural network systems like Chemprop, when integrated with efficient graph-based search structures such as hierarchical navigable small world (HNSW) indices, facilitate the retrieval of chemically plausible subsets for docking, eliminating the need for exhaustive brute-force evaluation of the complete library. Retrieval-augmented docking (RAD) is a hierarchical screening framework that utilizes molecular vectorization and approximate nearest-neighbor retrieval to minimize ultra-large compound libraries before implementing physics-based docking on the selected subset. This is regarded as an engineering layer for prioritization rather than a substitute for docking itself [219,220,221,222,223].

**Figure 6:**
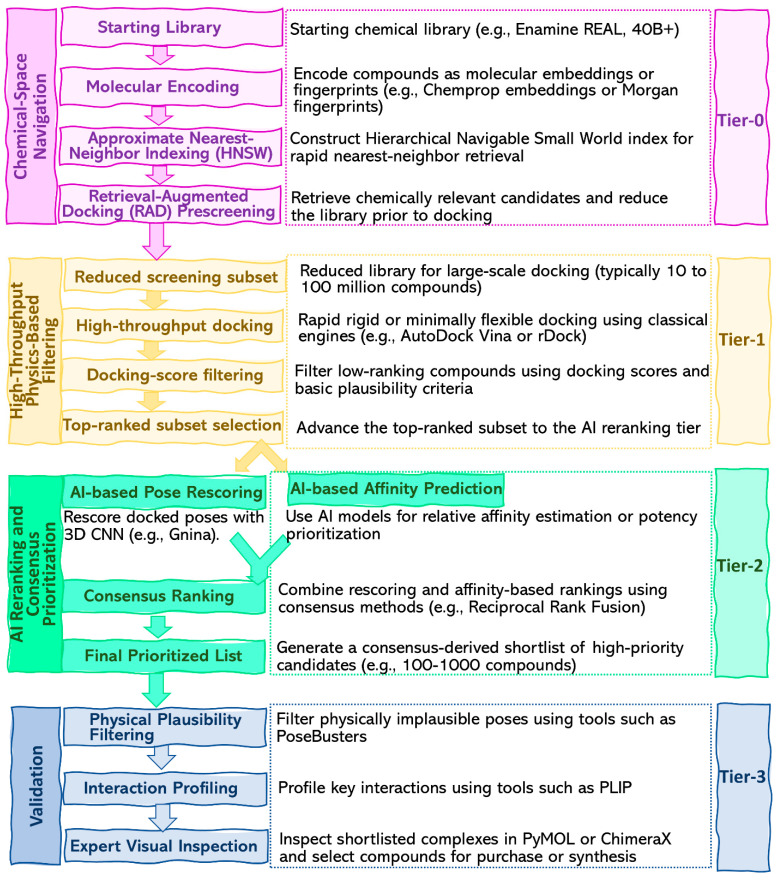
Tiered AI-augmented screening pipeline integrating chemical-space navigation, high-throughput physics-based docking, AI rescoring or affinity prediction, consensus ranking, and final validity and interaction-level inspection.

Practically, AI can be deployed at the Tier-0 stage to identify a manageable subset from an otherwise intractable library, allowing conventional high-throughput docking to proceed on the reduced set (Figure 6). This technique is especially appealing when the initial collection is chemically varied and synthetically attainable, yet excessively vast for comprehensive screening using local resources. The significance of RAD is not that it removes the need for energetic evaluation but that it concentrates docking effort on compounds that are more likely to occupy relevant regions of chemical space [219,222].

### 5.4. Physics-Based Filtering of AI-Prioritized Libraries

Following AI-based reduction in the screening library, a high-throughput physics-based layer remains essential. At this stage, rapid rigid or minimally flexible docking serves as the initial physically interpretable filter, eliminating compounds that are geometrically implausible or energetically unfavorable within the binding site. For standard protein targets, engines such as AutoDock Vina and rDock remain useful baseline platforms because they are computationally efficient, readily scriptable, and well established in large-scale screening workflows. In AI-augmented pipelines, their role is therefore not displaced but repositioned as the first energetically grounded filtering step applied after AI-driven library refinement [212].

Selection of the docking engine should remain guided by the underlying chemistry of the target. Metalloenzymes, in particular, often demand metal-aware treatment because standard scoring functions may fail to reproduce coordination geometries and electrostatic interactions with sufficient reliability. AutoDock4Zn is an example of a specialized extension designed for zinc-containing systems, and the broader docking literature continues to identify metalloproteins as a persistent challenge. In difficult cases, local quantum-mechanical or QM/MM validation may offer a more chemically faithful assessment of coordination geometry than classical scoring alone. By contrast, peptide binders present a different limitation: conventional small-molecule docking engines are often poorly suited to systems in which peptide flexibility and folding upon binding dominate the search landscape. Methods such as CABS-dock were developed specifically for this setting and permit flexible protein–peptide docking without the need to prespecify a binding site [160,224,225,226].

**Table 5:** AI-augmented tools for docking and structure-based drug design (SBDD).

SN	Tools	Stage	AI Paradigm	Typical Application	Reference/Official Link
1	Boltz-2	Complex structure/affinity prediction	Diffusion co-folding model	Pose generation; affinity scoring	[27] https://github.com/jwohlwend/boltz
2	CarsiDock	DL-guided docking	Pretrained deep learning-guided docking	Pose prediction/ranking	[227] https://github.com/carbonsilicon-ai/CarsiDock
3	Chai-1	Complex structure prediction	Multimodal foundation model	Pose/complex generation	[28] https://github.com/chaidiscovery/chai-lab
4	Deep Docking (DD protocol)	AI-accelerated virtual screening	QSAR/deep models trained on docking subsets to prune huge libraries	Billion-scale VS acceleration	[228] https://github.com/jamesgleave/DD_protocol
5	DeltaDock	Molecular docking	Unified deep learning framework	Docking and robust benchmarking	[229] https://github.com/jiaxianyan/DeltaDock
6	DiffBindFR	Flexible docking	SE(3)-equivariant diffusion framework	Flexible protein–ligand docking	[230] https://github.com/HBioquant/DiffBindFR
7	DiffDock	Pose prediction/blind docking	SE(3)-equivariant diffusion model	Pose generation and ranking	[231] https://github.com/gcorso/DiffDock
8	DynamicBind	Fully flexible complex prediction	Equivariant generative model/diffusion-style learning	Flexible protein–ligand complex modeling	[232] https://github.com/luwei0917/DynamicBind
9	EBMDock	Protein–protein docking	Differentiable energy-based model	Pose sampling/ranking	[233] https://github.com/wuhuaijin/EBMDock
10	EquiBind	Pose prediction/blind docking	SE(3)-equivariant geometric deep learning	Fast direct pose prediction	[234] https://github.com/HannesStark/EquiBind
11	FABind/FABind+	Pose prediction/blind docking	Geometric deep learning with improved pocket prediction	Fast blind docking	[235] https://github.com/QizhiPei/FABind
12	FlowDock	Generative docking + affinity prediction	Geometric flow matching	Joint structure and affinity modeling	[236] https://github.com/BioinfoMachineLearning/FlowDock
13	GNINA	Docking + rescoring	3D convolutional neural networks on atom grids	Drop-in docking engine/rescoring layer	[198,237] https://github.com/gnina/gnina
14	KarmaDock	Docking acceleration + pose generation + scoring	Deep learning model combining pose correction and strength estimation	High-throughput AI docking	[238] https://github.com/schrojunzhang/KarmaDock
15	NeuralPLexer	Complex structure prediction	Multiscale deep generative model	Protein–ligand structure prediction	[239] https://github.com/zrqiao/NeuralPLexer
16	Open-ComBind	Data-driven pose selection	Physics-based docking + learned cross-ligand consistency	Pose selection/affinity-related ranking	[240] https://github.com/drewnutt/open_combind
17	OpenDock	Docking framework with ML scoring	PyTorch framework with traditional and ML scoring functions	Method development and docking	[60] https://github.com/guyuehuo/opendock
18	OpenFold3-preview	Complex structure prediction	AF3-based co-folding model	Pose/complex generation	[241] https://github.com/aqlaboratory/openfold-3
19	Open-source DD protocol (optimized)	AI-accelerated virtual screening	Open implementation of deep docking workflow	Large-library pruning and analysis	[242] https://github.com/MichaelaBrezinova/open_source_deep_docking_protocol
20	PILOT (e3moldiffusion)	Pocket-conditioned multi-objective generation	Equivariant diffusion with guided generation	Generative SBDD/optimization	[243] https://github.com/pfizer-opensource/e3moldiffusion
21	Pocket2Mol	Pocket-conditioned de novo design	Equivariant autoregressive generative model	Hit generation inside pockets	[244] https://github.com/pengxingang/Pocket2Mol
22	PPDOCK	Blind docking	Pocket-prediction-based protein–ligand docking	End-to-end blind docking	[245] https://github.com/JieDuTQS/PPDOCK
23	RoseTTAFold All-Atom	Protein–ligand/complex prediction	All-atom deep structure model	Pose/complex generation	[217] https://github.com/baker-laboratory/RoseTTAFold-All-Atom
24	RTMScore	ML rescoring/scoring function	Graph transformer + residue-atom distance likelihood potential	Rescoring poses/affinity-related scoring	[246] https://github.com/sc8668/RTMScore
25	SampleDock	Generative-design + docking loop	Iterative generative model coupled to docking	Lead-generation workflow	[247] https://github.com/atfrank/SampleDock
26	SurfDock	Complex prediction/screening	Surface-informed diffusion model	Pose prediction and screening	[248] https://github.com/CAODH/SurfDock
27	TankBind	Pose + affinity prediction	Geometric deep learning on protein pocket/ligand graphs	Pose generation plus affinity estimation	[249] https://github.com/luwei0917/TankBind
28	TargetDiff	Pocket-conditioned de novo design	3D equivariant diffusion model	Generative SBDD/affinity-aware design	[250] https://github.com/guanjq/targetdiff
29	Uni-Mol (Docking)	3D representation learning for docking/SBDD	Large-scale 3D molecular + pocket pretraining	Binding conformation prediction and broader SBDD tasks	[25,251] https://github.com/deepmodeling/Uni-Mol

### 5.5. AI Rescoring, Affinity Prediction, and Consensus Ranking

Once a reduced set of docked complexes is available, the most natural role for AI is in rescoring and rank refinement. GNINA is the canonical open implementation of this strategy, incorporating convolutional neural-network (CNN) scoring into the docking workflow and showing improved virtual-screening performance relative to empirical scoring alone on several benchmark datasets. In this setting, AI does not supplant docking-based pose generation, but instead reevaluates candidate poses through learned spatial representations of protein–ligand contacts. This distinction is consequential, because CNN-based rescoring is generally most effective when applied to poses that have already satisfied a reasonable physics-based filter [237,252,253].

A second, more ambitious use of AI is affinity prediction. Recent foundation models aim not only to recover bound structures, but also to estimate quantitative binding strength directly from the modeled complex. Boltz-2 serves as a notable recent example; however, given its status as a preprint, its performance must be interpreted with caution until further external validation is conducted. These methods are therefore best framed as emerging tools for affinity-aware prioritization rather than as mature replacements for established binding assays or rigorously calibrated free-energy workflows. Moreover, consensus strategies can enhance ranking stability when multiple scoring outputs are present. Simple rank-fusion methods, such as reciprocal rank fusion, are particularly advantageous as they enable the integration of heterogeneous signals without necessitating direct normalization across fundamentally distinct scoring frameworks [27,254,255].

### 5.6. Validation of Physical Plausibility and Binding Interactions

AI-augmented SBDD should include a dedicated validity gate between model generation and final candidate nomination. This has become particularly important because high-ranking AI-generated poses can still violate fundamental stereochemical or steric constraints. PoseBusters offers a practical framework for this stage by assessing chemically and physically meaningful features of predicted complexes, and it was developed specifically in response to the tendency of several AI docking methods to produce poses that are visually persuasive yet physically invalid. More broadly, these findings highlight an important methodological point: ranking quality and physical plausibility are distinct criteria and must be evaluated independently [113,205].

Following physical filtering, interaction-level validation provides an additional layer of assessment. Tools such as PLIP can systematically annotate hydrogen bonds, hydrophobic contacts, salt bridges, metal interactions, and aromatic contacts in docked or predicted complexes, helping to determine whether retained poses are consistent with known pharmacophoric requirements or mechanistic expectations. This step is especially useful when AI rescoring and classical docking yield discordant results, because interaction profiling can help distinguish chemically sensible poses from those that are merely algorithmically preferred. At this stage, expert visual inspection in PyMOL or ChimeraX remains essential, particularly when the aim is to nominate compounds for synthesis or procurement rather than to maximize performance on a benchmarking task [112,113].

### 5.7. AI Strategies Across Distinct Target Classes

The utility of AI augmentation varies substantially across target classes. For standard proteins with high-quality holo structures, AI is often most effective downstream, particularly in rescoring, consensus ranking, and interaction-based triage. For apo or structurally uncharacterized targets, however, AI-based structure generation may serve as the enabling step for SBDD, provided that the resulting complexes are rigorously validated [256]. In metalloenzymes, AI may assist prioritization, but chemically specialized scoring and, when necessary, QM-informed refinement remain indispensable because metal coordination is still not captured reliably by generic learned models [225,257,258]. Likewise, for peptide binders, AI-generated starting complexes can be informative, but peptide-specific docking and refinement remain necessary because peptide conformational plasticity is poorly approximated by small-molecule frameworks [160,259].

A further limitation concerns binding-site type. Current evidence indicates that co-folding approaches are generally more dependable for canonical orthosteric binding than for allosteric recognition, where both site identification and pose recovery are often more challenging. Accordingly, in AI-augmented SBDD, co-folding should be treated as a hypothesis-generating approach when the site is uncertain, rather than as definitive proof of binding mode [29,260]. In these settings, pocket analysis, orthogonal docking, mutational evidence, and experimental validation remain particularly important.

### 5.8. Reporting Standards, Reproducibility, and Interpretation Limits

Because AI-augmented SBDD comprises multiple interdependent stages, reproducibility depends on transparent reporting across the full workflow. A rigorous study should clearly document how the target structure was obtained or generated; whether templates, ligands, multiple-sequence alignments (MSAs), or restraints were supplied to the predictive model; how the chemical library was represented and reduced; which docking engine and associated parameters were employed; whether AI-based rescoring or affinity prediction was applied; how consensus ranking was conducted; and which physical or interaction-level filters were imposed before final candidate nomination. Without this level of reporting, cross-study comparisons remain difficult to interpret, because observed gains may derive from differences in data curation, target preparation, or triage strategy rather than from the AI methodology itself [22,205,261,262].

More broadly, AI-augmented SBDD should be regarded as a framework for prioritization rather than as proof of binding mode or biological efficacy. The current generation of models has undoubtedly expanded the scope of feasible structure prediction and docking tasks, yet benchmark studies continue to expose persistent weaknesses in generalization to novel sequences, multiligand assemblies, chemically challenging targets, and the production of physically valid poses. AI is therefore most powerful when used as a layered accelerator that reduces search space, improves ranking, and enhances hypothesis generation, while remaining firmly coupled to chemistry-aware docking, plausibility checks, and experimental validation [43,113,205,263].

## 6. Challenges and Opportunities

Open availability does not automatically eliminate infrastructure and interoperability barriers. Although open-source tools substantially reduce licensing costs, they do not fully remove practical asymmetries in access. This is increasingly evident in AI-enhanced SBDD workflows, where computational demand can shift the bottleneck from software cost to hardware availability. For example, AutoDock-GPU explicitly targets CUDA- and OpenCL-enabled accelerators and reports substantial speedups relative to serial AutoDock4, while GNINA integrates convolutional neural-network scoring and documents GPU-dependent operating modes, with some CNN workflows becoming markedly more expensive when used beyond simple rescoring. Similarly, recent evaluations of co-folding methods such as RoseTTAFold All-Atom [33,217] and Boltz-1 [218] have been conducted on A100 80 GB hardware, underscoring that some openly available structure-prediction workflows remain demanding to deploy routinely at scale. From this perspective, the distinction between open and easily deployable remains significant. A method may be openly licensed and scientifically valuable while nevertheless remaining difficult to reproduce in resource-constrained environments owing to requirements for specialized accelerators, substantial memory, or complex systems integration.

Interoperability remains a second, closely related challenge. Contemporary open-source SBDD workflows rarely rely on a single program; instead, they typically combine cheminformatics libraries, preparation tools, docking engines, rescoring models, and post-docking analysis utilities. This has improved markedly with the availability of interoperability-focused tools such as Open Babel for file-format conversion and cheminformatics processing, Meeko for molecular parameterization and docking-oriented software interoperability, and PLIP [112] for structured interaction profiling suitable for downstream processing. However, the very need for such bridging tools also highlights that workflow exchange is still not frictionless across groups. More broadly, reproducibility studies in computational drug discovery continue to emphasize that workflows, pipelines, and research documentation are essential for making multistage analyses reusable, while recent open-science perspectives in cheminformatics stress the importance of harmonized data formats and robust exchange platforms. Taken together, these developments suggest that the key challenge is no longer the absence of open tools, but the absence of uniformly adopted, low-friction standards for connecting mature tools into transparent, portable, and well-documented scientific workflows. The opportunity, therefore, lies not only in developing new methods but also in standardizing interfaces, workflow metadata, and reporting practices so that open pipelines become easier to exchange, audit, and reproduce across laboratories.

## 7. Future Perspectives

One of the most important future priorities for open-source SBDD will be to reduce the gap between formal software openness and real-world deployability. While source-code availability remains a foundational requirement, it does not in itself guarantee that methods can be installed, executed, and reproduced reliably across heterogeneous research settings. Addressing this gap will require sustained emphasis on hardware-efficient inference, containerized and reproducible software distribution, richer workflow metadata, and more robust interoperability standards linking ligand and receptor preparation, docking, rescoring, and post-docking analysis.

More fundamentally, the next phase of progress is likely to depend on a transition from tool-centered innovation to ecosystem-level sustainability. For open-source docking to achieve long-term maturity, maintenance must be recognized as a core component of research infrastructure rather than as an informal activity that follows initial publication. This requires more durable governance models, clearer ownership structures, and funding mechanisms capable of extending beyond the lifespan of individual grants, trainees, or laboratories. In this context, a central lesson is that future progress will depend not only on the introduction of new scoring functions, search algorithms, or artificial intelligence modules, but also on the sustained maintenance, validation, documentation, and stewardship of the tools that already underpin the field.

A second important direction concerns formal succession planning and modular renewal. The docking ecosystem already offers several constructive examples in which replacement has occurred through redesign rather than abrupt obsolescence. Meeko [97] has increasingly displaced MGLTools-based preparation workflows, pydock3 has emerged as a successor to DOCK Blaster and blastermaster [264], and AutoDock Vina has continued to evolve through Python bindings, batch-processing capabilities, and active release cycles [132,157]. Together, these developments suggest a healthier model in which software is restructured into interoperable layers rather than preserved indefinitely as closed or aging monoliths. An important future opportunity is to institutionalize this practice more explicitly by encouraging software projects to define transparent deprecation policies, identify recommended successors, document compatibility boundaries, and provide migration guides so that users can move across software generations without rebuilding workflows from scratch.

Usability-by-design should also be regarded as a strategic priority. Open-source docking will expand its scientific reach only if it becomes easier for non-specialist users to install, operate, interpret, and audit. Recent developments already point in this direction. MolModa illustrates one pathway through browser-based accessibility [184], whereas AutoDock Vina [18,132] and GNINA [198] illustrate another through improved documentation, official binaries, and container-oriented deployment. Future progress should therefore extend beyond methodological sophistication alone and include clearer default workflows, more robust installation pathways, better tutorials and worked examples, and a stronger focus on the practical experience of new users. Within open-source SBDD, accessibility is no longer a secondary concern; it is a prerequisite for wider adoption, community growth, and durable maintenance.

Finally, the long-term health of open-source docking will depend on managed diversity rather than unchecked proliferation. Software forks are likely to remain an important source of experimentation and innovation, but their enduring value depends on transparent documentation, visible maintenance status, sound governance, and interoperability with the broader ecosystem. The most productive software lineages are not those that merely generate multiple variants, but those that make the relationships among parent tools, forks, and successors intelligible to users. The histories of rDock [265] and RxDock [151,266], as well as Vina [156,158,267], smina [153], and GNINA [253], illustrate that the next stage of maturity will require better curation of project lineages, richer metadata, and clearer signals regarding which tools are legacy systems, which are transitional, and which remain actively supported. Open-source docking will be strongest when innovation through diversity remains possible, while maintenance status, migration pathways, and interoperability are made explicit rather than left for users to infer.

## 8. Conclusions

In conclusion, open-source molecular docking and AI-augmented SBDD are rapidly evolving from collections of individual tools into steadily integrated and methodologically sophisticated discovery pipelines. Despite significant limitations, especially regarding affinity ranking, receptor-state uncertainty, chemical-state assignment, and benchmark realism, the field has evidently advanced beyond mere low-cost alternatives to commercial software. The current strength is found in the increasing capacity to integrate transparent preparation protocols, scalable docking engines, machine-learning-assisted rescoring, emerging co-folding strategies, and reproducible validation schemes within fully auditable workflows. The future influence of this ecosystem will rely not on the ability of any single method to address all facets of molecular recognition, but on the effectiveness of integrating, benchmarking, and reporting complementary approaches. As methodological standards advance, open-source and AI-enabled SBDD will increasingly contribute to enhancing the rigor, accessibility, and reproducibility of computational drug discovery.

## Figures and Tables

**Table 1:** Representative prospective hit-discovery studies using free or academically accessible docking/SBDD workflows.

SN	Study	Target	Workflow	Tested/Hit Rate	Best Potency/Validation
1	Cabeza de Vaca et al. 2026 [48]	GPR139	Docking 235 million compounds to the GPR139 binding site	68 top-ranked compounds tested; 5 full agonists (7.4%)	Potencies ranged from 160 nM to 3.6 µM; optimized compounds showed in vivo behavioral effects; cryo-EM confirmed the predicted binding mode
2	Tummino et al. 2025 [49]	CB1 receptor	Docking 74 million tangible molecules against human CB1 receptor	46 tested; 9 active by radioligand competition (19.6%)	Optimization yielded ‘1350, a Ki 0.95 nM full agonist; analgesic activity at 0.05 mg/kg; cryo-EM confirmed the pose
3	Zhou et al. 2024 [44]	KLHDC2; NaV1.7	RosettaVS AI-accelerated virtual screening of multi-billion libraries	KLHDC2: 7 hits (14%); NaV1.7: 4 hits (44%)	All hits were single-digit µM; X-ray validated the KLHDC2 docking pose
4	Díaz-Holguín et al. 2024 [50]	TAAR1	AlphaFold-guided docking of >16 million compounds, compared with a homology model screen	AF2 screen: 30 tested; 18 agonists (60%); homology model: 32 tested; 7 agonists (22%)	Agonists ranged from 12 to 0.03 µM; one lead showed antipsychotic-like effects in wild-type but not TAAR1-knockout mice
5	Liu et al. 2024 [52]	Calcium-sensing receptor (CaSR)	Large-library docking of 2.7 million and 1.2 billion molecules against CaSR	2.7 M screen: 13.6% hit rate; 1.2 B screen: 36.5% hit rate	Docking produced hits up to 37-fold more potent; optimization yielded nanomolar leads, and one lead lowered serum parathyroid hormone in mice
6	Lyu et al. 2024 [43]	σ2 receptor; 5-HT2A receptor	DOCK3.8 prospective docking against AlphaFold2 models versus experimental structures; >490 million (σ2) and 1.6 billion (5-HT2A) molecules	σ2: AF2 55% vs. experimental 51% at 1 µM; 5-HT2A: AF2 26% vs. experimental 23% at 10 µM	σ2 AF2 hits had Ki 1.6–84 nM; 5-HT2A AF2 agonists had EC50 42 nM–1.6 µM; cryo-EM of Z7757 supported the docking pose
7	Luttens et al. 2025 [51]	OGG1	Docking of 14 million fragment-like molecules and 235 million lead-like molecules against OGG1	29 top-ranked compounds tested; 4 binders (13.8%)	X-ray crystallography confirmed docking poses; fragment elaboration yielded submicromolar inhibitors with cellular anti-inflammatory and anticancer effects
8	Gahbauer et al. 2023 [47]	EP4R	Docked 440 million compounds against an EP4R using DOCK3.7	71 top-ranked compounds tested; 6 (8.5%) dose-dependent antagonists	Best initial hit had IC50 850 nM; optimization reached Ki 16 nM
9	Kaplan et al. 2022 [46]	5-HT2A receptor	DOCK-based screening of 75 million tetrahydropyridines against a receptor model	17 tested; 4 initial low-µM actives (23.5%)	(R)-69 and (R)-70 reached EC50 41 nM and 110 nM; cryo-EM confirmed the predicted binding mode
10	Everson et al. 2021 [53]	Plasmodium falciparum HSP90	AutoDock/Smina screening of 13 million ZINC15 compounds	12 tested; 3 active compounds (25.0%)	Best hit had EC50 0.98 µM
11	Stein et al. 2020 [45]	MT1 melatonin receptor	Docking >150 million virtual molecules against an MT1 crystal structure	38 synthesized/tested; 15 active (39%)	Ligands ranged from 470 pM to 6 µM; selective MT1 inverse agonists showed in vivo circadian effects in mice

Abbreviations: AF2 = AlphaFold2; CB1 = cannabinoid-1; EC50 = half-maximal effective concentration; EP4R = prostaglandin E2 receptor 4; GPR139 = G protein-coupled receptor 139; IC50 = half-maximal inhibitory concentration; Ki = inhibition constant; TAAR1 = trace amine-associated receptor 1; OGG1 = 8-oxoguanine DNA glycosylase; CaSR = calcium-sensing receptor.

**Table 3:** Open-source installable software for docking and structure-based drug design (SBDD).

SN	Tools	Stage	Primary Function	Typical Application	Reference/Official Link
1	AMDock	Graphical docking assistant	GUI workflow for AutoDock4/Vina plus preparation helpers	Guided preparation, box definition, docking	[128] https://github.com/Valdes-Tresanco-MS/AMDock
2	ATTRACT	Macromolecular docking suite	Coarse-grained rigid-body/flexible docking	Macromolecular docking and refinement	[129,130] https://github.com/sjdv1982/attract
3	AutoDock CrankPep (ADCP)	Peptide docking engine	Monte Carlo peptide folding + AutoDock affinity grids	Peptide docking	[131] https://github.com/ccsb-scripps/ADCP
4	AutoDock Vina	General-purpose small-molecule docking engine	Gradient-based local optimization + Vina/Vinardo/AD4 scoring support	Routine docking and virtual screening	[18,132] https://github.com/ccsb-scripps/AutoDock-Vina
5	AutoDock4	Classical small-molecule docking engine	Lamarckian genetic algorithm + empirical free-energy scoring	Docking/redocking/VS baseline	[85] https://github.com/ccsb-scripps/AutoDock4
6	AutoDockFR (ADFR)	Flexible-receptor docking	Genetic algorithm with explicitly specified receptor flexibility	Flexible-side-chain docking	[133,134] https://ccsb.scripps.edu/adfr/
7	AutoDock-GPU	Accelerated AutoDock4 implementation	GPU/OpenCL/CUDA/SYCL acceleration of AutoDock4 search	Large-scale VS on accelerators	[135] https://github.com/ccsb-scripps/AutoDock-GPU
8	DOCK 6	Classical docking suite	Sphere matching/anchor-and-grow/GA/grid scoring options	Docking, de novo design, rescoring	[136] https://dock.compbio.ucsf.edu/DOCK_6/index.htm
9	Dockey	Integrated docking GUI/workbench	Pipeline integrating preparation, docking, interaction detection, visualization	Large-scale docking and vs. from GUI	[137] https://github.com/lmdu/dockey
10	DockingPie	Consensus docking PyMOL plugin	GUI integration of Vina, smina, ADFR, RxDock	Consensus docking and result analysis	[138] https://github.com/paiardin/DockingPie
11	DockoMatic	HTVS GUI manager	AutoDock job creation/management automation	Batch job setup and management	[139,140,141] https://sourceforge.net/projects/dockomatic/
12	FlexAID	Flexible docking engine, NRGsuite PyMOL plugin	Genetic algorithm + soft surface complementarity scoring	Flexible docking, non-native receptor cases	[142] https://github.com/NRGlab/FlexAID
13	HADDOCK3	Integrative biomolecular docking platform	Information-driven docking with restraints and modular workflows	Integrative docking with prior data	[120] https://github.com/haddocking/haddock3
14	Idock	Multithreaded docking tool	Vina-inspired search optimized for speed	Fast virtual screening	[143] https://github.com/gloglita/idock
15	LeDock	Small-molecule docking engine	Fast flexible docking with empirical scoring	Rapid protein–ligand docking and VS	https://www.lephar.com/software
16	LightDock	Macromolecular docking framework	Glowworm Swarm Optimization (GSO)	Protein–protein docking	[144,145] https://github.com/lightdock/lightdock
17	MetalDock	Metal-complex docking tool	Python workflow for docking metal-organic compounds	Dock organometallic compounds to proteins/DNA/biomolecules	[146] https://github.com/MatthijsHak/MetalDock
18	MzDOCK	Automated GUI based pipeline for Molecular Docking	Integrates docking, ligand preparation, visualization, and post-docking analysis in a single GUI environment	Protein-ligand docking and post-docking analysis	[147] https://github.com/Muzatheking12/MzDOCK
19	OpenDock	Extensible docking framework	Traditional + machine-learning scoring functions in a PyTorch framework	Method development, docking, rescoring	[60] https://github.com/guyuehuo/opendock
20	pydock3	Automation/wrapper for DOCK3 pipeline	Python orchestration around UCSF DOCK	Automated DOCK3 campaigns, parameter optimization	[148] https://github.com/docking-org/pydock3
21	PyRx	GUI virtual screening workbench	Front-end around AutoDock/Vina and preparation utilities	Teaching, small/medium screening campaigns	[149] https://pyrx.sourceforge.io/
22	QuickVina 2	Fast Vina derivative	Heuristic acceleration of Vina search	Rapid docking/scrseening	[150] https://github.com/QVina/qvina
23	QuickVina-W	Blind-docking-oriented Vina derivative	QVina2 acceleration + thread communication for wider boxes	Blind docking in wide search spaces	[71] https://qvina.github.io/
24	rDock	HTVS-oriented docking engine	Stochastic search with cavity maps and scoring for proteins/nucleic acids	HTVS and binding-mode prediction	[151] https://github.com/CBDD/rDock
25	SEED	Fragment docking program	Force-field/solvation-based exhaustive fragment docking	Fragment docking and fragment-based screening	[152] https://gitlab.com/CaflischLab/SEED
26	smina	Vina fork for scoring/minimization	Vina-based search with custom scoring support	Custom scoring-function development and minimization	[153] https://github.com/mwojcikowski/smina
27	Uni-Dock	GPU-accelerated docking engine	GPU implementation supporting vina/vinardo/ad4 scoring	Ultra-fast virtual screening	[154] https://github.com/dptech-corp/Uni-Dock
28	Vina-Carb	Specialized Vina derivative	Vina modified for carbohydrate torsional preferences	Glycoligand docking	[155] https://github.com/Alicecomma/VinaCarb
29	Vina-GPU	GPU-accelerated Vina derivative	Large-scale docking acceleration	Speedups for Vina workflows	[156,157,158] https://github.com/DeltaGroupNJUPT/Vina-GPU-2.1
30	VinaXB	Specialized Vina derivative	Vina with explicit halogen-bond scoring term	Halogen-sensitive docking	[159] https://github.com/sirimullalab/vinaXB

**Table 4:** Open-source webservers for docking and structure-based drug design (SBDD).

SN	Tools	Stage	Primary Function	Typical Application	Reference/Official Link
1	CABS-dock	Flexible peptide docking	Binding-site search with fully flexible peptide docking	Peptide docking without prior site knowledge	[160] https://biocomp.chem.uw.edu.pl/CABSdock
2	CB-Dock2	Blind-docking server	Automatic cavity detection + docking + homologous template fitting	Very accessible blind docking	[20] https://cadd.labshare.cn/cb-dock2/
3	ClusPro	Protein–protein docking	FFT-based global sampling with clustering-driven model selection	Accessible macromolecular docking and clustering-based ranking	[161] https://cluspro.org/
4	DINC-Ensemble	Ensemble docking	Incremental docking of large ligands against receptor conformations	Large-ligand docking and ensemble docking	[132,162,163] https://dinc-ensemble.kavrakilab.rice.edu/
5	DockThor/DockThor-VS	Docking and VS	DockThor engine with web-based submission	Docking and virtual screening	[42,164] https://dockthor.lncc.br/v2/
6	EDock	Protein–ligand docking	Based on replica-exchange Monte Carlo simulations for blind docking	Docking	[165] https://aideepmed.com/EDock/
7	GalaxyWEB	Multi-tool docking/modeling suite	Structure prediction, refinement, docking, target prediction	Protein–ligand, protein–peptide, and protein–protein docking; Compound target prediction; Covalent ligand docking	[166,167,168,169,170,171,172,173,174,175] https://galaxy.seoklab.org/
8	GRAMM Web	Macromolecular docking	Free-docking and template-based docking modes for protein complexes	Protein–protein docking	[176] https://gramm.compbio.ku.edu/gramm
9	HADDOCK web portal	Integrative docking portal	HADDOCK workflows via web portal	Restraint-driven online docking	[177,178] https://alcazar.science.uu.nl/
10	HawkDock	Protein–protein docking and reranking	ATTRACT-based sampling with HawkRank and MM/GBSA reranking	Protein–protein docking and ranking	[179] https://cadd.zju.edu.cn/hawkdock/
11	HDOCK	Hybrid template	Template-based modeling + ab initio docking	Macromolecular docking	[180,181,182] http://hdock.phys.hust.edu.cn/
12	HPEPDOCK	Blind peptide docking	Hierarchical protein–peptide docking algorithm	Protein–peptide docking	[183] http://huanglab.phys.hust.edu.cn/hpepdock/
13	MolModa	Browser-based docking environment	Web-based end-to-end molecular docking workflow	Interactive preparation, docking, and visualization	[184] https://github.com/durrantlab/molmoda
14	MTiAutoDock/MTiOpenScreen	Docking + screening	AutoDock-based site-specific/blind docking and virtual screening	Docking and library screening	[185] https://bioserv.rpbs.univ-paris-diderot.fr/services/MTiOpenScreen/
15	NPDock	Protein–nucleic acid docking	GRAMM-based global docking plus scoring, clustering, and refinement	RNA–protein and DNA–protein docking	[123] https://genesilico.pl/NPDock/
16	ProteinsPlus	Protein-structure analysis and structure-based molecular design	Integrated protein-structure analysis and drug-design platform with tools for docking, binding-site analysis, protonation, visualization, and structural profiling	Protein structure analysis, binding-site/druggability assessment, and automated protein-ligand docking	[186] https://proteins.plus/
17	pyDockDNA	Protein–DNA docking	Energy-based pyDockDNA scoring/workflow	Protein–DNA complex modeling	[187] https://model3dbio.csic.es/pydockdna/
18	pyDockWEB	Protein–protein docking	FTDock sampling + pyDock scoring	Rigid-body macromolecular docking	[188] https://life.bsc.es/pid/pydockweb
19	ROSIE	Multi-step SBDD platform	Rosetta-based modeling and docking	Protein docking, Protein–ligand docking, Peptide docking/refinement	[189,190] https://rosie.graylab.jhu.edu/
20	SeamDock	Collaborative online docking	Common web framework wrapping several docking tools	Teaching/collaborative docking	[41,191] https://bioserv.rpbs.univ-paris-diderot.fr/services/SeamDock/
21	SwissDock	Protein–small-molecule docking	Current server supports attracting-cavities and AutoDock Vina engines	Docking	[5,21] https://www.swissdock.ch/
22	VSTH (MatGen Virtual Screening)	Integrated structure-based virtual-screening platform	Protein preparation, pocket selection, docking (AutoDock Vina, AutoDock4, GalaxyDock3, iDock, iGemdock, and LeDock), monitoring, and analysis	End-to-end structure-based virtual screening	[192] https://matgen.nscc-gz.cn/VirtualScreening.html
23	Webina	Docking	Browser-based AutoDock Vina docking	Docking	[193] https://github.com/durrantlab/webina
24	ZDOCK	Protein docking server	Automatic rigid-body protein docking	Protein–protein docking	[194] https://zdock.wenglab.org/

## Data Availability

No new data were created or analyzed in this study. Data sharing is not applicable to this article.
